# A study of medication safety in rural older people in Luzhou City

**DOI:** 10.3389/fpubh.2025.1656427

**Published:** 2026-01-14

**Authors:** Shiying Xu, Panwen Gong, Ping Huang, Liang Liu, Xiuli Liu, Ting Liu, Jingying Li

**Affiliations:** 1 Department of Pharmacy, Luzhou People's Hospital, Luzhou, Sichuan, China; 2 Department of Urology, The First Affiliated Hospital of Chongqing Medical University, Chongqing, China; 3 Department of Child Healthcare, Luzhou People's Hospital, Luzhou, Sichuan, China

**Keywords:** medication safety, older people, questionnaire survey, rational drug use, rural areas

## Abstract

**Objective:**

To assess the current status and determinants of medication safety among rural older residents in Luzhou City, with the aim of informing targeted strategies to promote safe and rational medication use.

**Methods:**

A cross-sectional study was conducted involving 100 older adults (≥60 years) and 60 primary care physicians from rural Luzhou. Data collection included structured questionnaires on medication use, adherence, and safety knowledge, as well as a review of 100 outpatient electronic medical records (EMRs) for chronic disease management. Prescription rationality was evaluated using the Medication Appropriateness Index (MAI), and potentially inappropriate medications (PIMs) were identified according to the 2023 American Geriatrics Society Beers Criteria. Descriptive statistics and chi-square tests were applied to examine associations between sociodemographic factors and unsafe medication practices.

**Results:**

Polypharmacy (≥3 concurrent medications) was reported by 72% of older participants, with 81% taking antihypertensive or cardiovascular drugs. Unsafe practices were common: 47% altered medication timing or missed doses, and 37% self-adjusted dosages. Knowledge gaps were evident, as only 43% consulted pharmacists about dosing or precautions, and just 23% would discontinue a drug and seek help if experiencing an adverse reaction. Lower education and household income were significantly associated with unsafe behaviors (*p* = 0.014). Among EMRs reviewed, 2% of prescriptions were rational, and 12% contained PIMs, most frequently long-acting benzodiazepines and NSAIDs.

**Conclusion:**

Rural older people in Luzhou face significant medication safety challenges, driven by high rates of polypharmacy, unsafe self-management behaviors, and socioeconomic disparities. Interventions should combine patient education, improved physician–patient communication, and systematic prescription review to reduce inappropriate medication use and related adverse outcomes.

## Introduction

The World Health Organization (WHO) and Chinese national policy define older individuals as those aged 60 years and above, a classification widely applied in public health planning and geriatric research. This threshold is particularly relevant in China, where demographic shifts have resulted in a rapidly aging population.

According to the Seventh Population Census of China in 2020, individuals aged ≥60 years account for 18.70% of the national population, with rural areas showing an even higher proportion at 23.81% (1). In Luzhou City alone, over 970,000 people are aged 60 or older, most of whom suffer from multiple chronic conditions. As these individuals require prolonged use of various medications, the issue of medication safety has become a critical public health concern. Prior studies, both internationally and within China, have identified medication safety as a persistent challenge for older adults, especially in the context of polypharmacy, low health literacy, and fragmented primary care (2, 3). Research in urban and eastern regions of China has demonstrated high rates of potentially inappropriate medications (PIMs), poor adherence, and unsafe self-medication practices among the older people (4). However, much of this evidence overlooks rural and under-resourced areas in western China, where the healthcare infrastructure is less developed and sociodemographic disadvantages—such as low educational attainment and household income—are more pronounced. Furthermore, there is limited understanding of how rural primary care physicians manage prescribing for older patients, and few studies have examined the combined roles of patient behavior and provider practices in contributing to unsafe medication use. These gaps highlight an urgent need for targeted, region-specific research to guide interventions that can enhance medication safety and address health disparities in vulnerable rural older populations (5).

Older populations, particularly in rural areas, are at heightened risk of adverse drug reactions (ADRs) due to factors such as polypharmacy, limited health literacy, and inadequate healthcare infrastructure (6). These risks are compounded by low educational attainment, limited access to healthcare services, and the absence of younger family members to assist with medication management (7, 8). Many older individuals struggle to understand drug labels, follow medication instructions, or recognize potential side effects, making them vulnerable to inappropriate medication use (9, 10). Consequently, inappropriate medication practices—such as altering dosages without medical advice, missing doses, or self-purchasing medications without a prescription—are common and can lead to poor health outcomes (11, 12).

While previous research has examined medication use among urban or institutionalized older people, there is limited evidence on rural older populations in western China (10). This gap is especially relevant for Luzhou, given its distinct demographic, educational, and healthcare access profile. Therefore, this study aims to investigate medication safety among rural older adults in Luzhou and assess the role of primary healthcare physicians in promoting safe medication use, with the ultimate goal of informing targeted interventions to reduce inappropriate medication use and improve health outcomes.

## Materials and methods

### Study population

This cross-sectional observational study was approved by the Ethics Committee of Luzhou People’s Hospital. The research adhered strictly to the principles of the Declaration of Helsinki, and written informed consent was obtained from all participants. The sample studied in this exploratory cross-sectional study consisted of approximately 100 seniors and 60 primary healthcare physicians from rural Luzhou City, Sichuan Province, China.

Setting an age threshold of ≥60 years was based on national demographics, as well as the World Health Organization’s (WHO) definition of older individuals in both clinical and public health policy contexts, globally and in China, for all individuals aged 60 years and older (13).

The older people sampling criteria specified seniors aged 60 years and older, residing in a rural area of Luzhou, with no cognitive impairment, and the ability to communicate verbally. Exclusion criteria were based on cognitive impairment or psychiatric disorders (e.g., Alzheimer’s disease) and for seniors older than 90 years.

Older participants in the study were recruited through convenience sampling from among eligible individuals in local community health centers and rural clinics. Village doctors or health workers explicitly advised seniors about the study, and allied local health workers invited eligible health workers to participate voluntarily.

For primary healthcare physicians, the inclusion criteria were to be a licensed primary healthcare physician (as opposed to non-licensed healthcare professionals) working as a physician providing primary care to seniors in rural Luzhou for at least one-year, prescribing medications, and regularly seeing seniors. Physicians working in a non-clinical capacity at the clinic or hospital facilities, agents with incomplete surveys, and physicians who are not primary care providers and were not involved in the direct care of seniors were excluded from the study.

In addition, a total of 100 electronic medical records (EMRs) from outpatient visits were reviewed at Luzhou People’s Hospital between January 1 and June 30, 2023. The prescribed EMRs were for the chronic conditions, including but not limited to hypertension, diabetes, or cardiovascular disease. The included EMRs had to represent individuals aged 60 years and older and contain all the details of the prescription, including the name of the medication, dosage, and duration of treatment. Both mental disorders classified medications, regardless of whether used for other medical conditions, and prescriptions with missing or fuzzy details, emergency-only EMRs from the illness classification group, and duplicate EMRs were excluded from the final analysis.

### Sample size justification

The sample sizes of 100 rural older participants and 60 primary healthcare physicians were determined based on feasibility and the exploratory nature of the study. As this was a cross-sectional, descriptive study aimed at identifying general patterns and potential risk factors for unsafe medication practices, a formal power calculation was not conducted. However, the selected sample was sufficient to estimate proportions with reasonable precision (e.g., for a prevalence estimate of 50%, a sample size of 100 yields a 95% confidence interval width of approximately ±10%). For subgroup comparisons (e.g., by education or income level), the sample provided enough statistical power to detect medium effect sizes using chi-square tests. While the findings are not intended to be generalizable to all rural regions in China, the sample is reflective of the local older population in Luzhou and is appropriate for generating hypotheses to guide future large-scale, multi-center studies.

### Survey instruments

Two structured questionnaires were created and implemented for the study: the “Luzhou Rural Older People Medication Safety Survey” (for older people) and the “Luzhou Primary Healthcare Physicians Medication KAP Survey” (for physicians) (14). The older people survey had domains about polypharmacy (concurrent use of three or more medications), medication adherence, self-medication behaviors, and knowledge of medication safety. The physician survey included domains related to the physicians’ knowledge, attitudes, and practices regarding the use of drugs in the older people.

The research instruments were pre-tested with small samples of 10 older people and five physicians to assess clarity and reliability. Internal consistency was sufficient, with Cronbach’s alpha values of 0.78 for the older people survey and 0.81 for the physician KAP survey. The instruments were not validated through large-scale psychometric testing; however, both instruments were adapted from previously established formats used in other geriatric medication studies.

Sociodemographic data were also collected from older participants, including age, gender, education, marital status, household income, and a history of chronic diseases, to explore possible relationships with medication behaviors using these sociodemographic data.

Medication rationality in the electronic medical records (EMRs) was assessed using the Chinese version of the Medication Appropriateness Index (MAI) (13) and the 2023 American Geriatrics Society Beers Criteria (15). The MAI has a scoring system ranging from 0 to 18, where higher scores indicate worse drug appropriateness. A score of ≥10 is irrational drug use. Potentially inappropriate medications (PIMs) were identified using the Beers Criteria and focused on inappropriate drugs for older patients, drug-disease interactions, and therapeutic duplications.

While internal consistency of the instruments was assessed through Cronbach’s alpha (0.78 for the older people survey and 0.81 for the physician survey), other psychometric properties such as content validity, construct validity, and test–retest reliability were not formally evaluated. This limitation reflects the exploratory nature of the study and constraints related to time and resources. However, both instruments were adapted from previously published and contextually relevant surveys used in geriatric medication research, and were pre-tested for clarity and comprehension among small pilot samples. Future research should aim to undertake full-scale validation procedures to strengthen measurement rigor.

Given the use of self-reported data from older participants and physicians, we acknowledge the potential for recall bias and social desirability bias. To minimize these effects, trained data collectors provided standardized explanations and administered surveys in a private setting, emphasizing that participation was anonymous and voluntary. Respondents were assured that their answers would remain confidential and would not affect their medical care or employment. Questionnaires were carefully worded in a neutral and nonjudgmental tone to reduce the likelihood of participants overreporting positive behaviors or underreporting undesirable ones. While these steps likely reduced bias to some extent, the possibility of residual response bias cannot be ruled out and is acknowledged as a study limitation.

### Statistical analysis

All survey and EMR data were analyzed using IBM SPSS Statistics version 26.0. Descriptive statistics (means, standard deviations, frequencies, and percentages) were used to summarize demographic variables, medication use patterns, and questionnaire responses. Chi-square tests and independent t-tests were applied to examine associations between medication safety behaviors and sociodemographic characteristics (e.g., education level, household income). To explore the potential influence of confounders, subgroup analyses were conducted by stratifying key variables (e.g., education levels, chronic disease presence). However, due to the limited sample size and the exploratory intent of the study, we did not conduct multivariate regression modeling or apply formal corrections for multiple comparisons (e.g., Bonferroni adjustment). As such, findings should be interpreted with caution, particularly where *p*-values approach conventional significance thresholds.

## Results

A total of 100 rural older adults participated in the study (Table 1). The sample included 52 males (52.0%) and 48 females (48.0%), with ages ranging from 60 to 89 years: 34 (34.0%) were aged 60–69 years, 41 (41.0%) were 70–79 years, and 25 (25.0%) were 80–89 years. Educational attainment was low for most participants, with 62 (62.0%) having no formal education or only primary school, 28 (28.0%) completing middle school, and 10 (10.0%) graduating from high school or higher. Sixty-five (65.0%) were married, 35 (35.0%) were widowed or single, 78 (78.0%) reported at least one chronic disease, and 66 (66.0%) had household incomes below the regional average. Sociodemographic data for the older participants are presented in Table 1.

**Table 1:** Sociodemographic characteristics of rural older participants (*n* = 100).

Characteristic	Category	*n*	%
Gender	Male	52	52
	Female	48	48
Age group (years)	60–69	34	34
	70–79	41	41
	80–89	25	25
Education level	No education/Primary school	62	62
	Middle school	28	28
	High school or higher	10	10
Marital status	Married	65	65
	Widowed/Single	35	35
Chronic disease	Yes	78	78
	No	22	22
Household income	Below regional average	66	66
	Above regional average	34	34

Polypharmacy was prevalent: In total, 72 participants (72.0%) met the definition of polypharmacy (≥3 medications used concurrently). The most common drug classes among all participants were antihypertensive/cardiovascular agents (*n* = 81, 81.0%), hypoglycemic agents (*n* = 34, 34.0%), and musculoskeletal medications (*n* = 13, 13.0%).

Knowledge of safe medication use was variable. Only 43 (43.0%) consulted pharmacists regarding dosage and precautions, 60 (60.0%) knew what an adverse drug reaction (ADR) was, and just 23 (23.0%) stated they would discontinue a drug and seek help if an ADR occurred.

**Figure 1:**
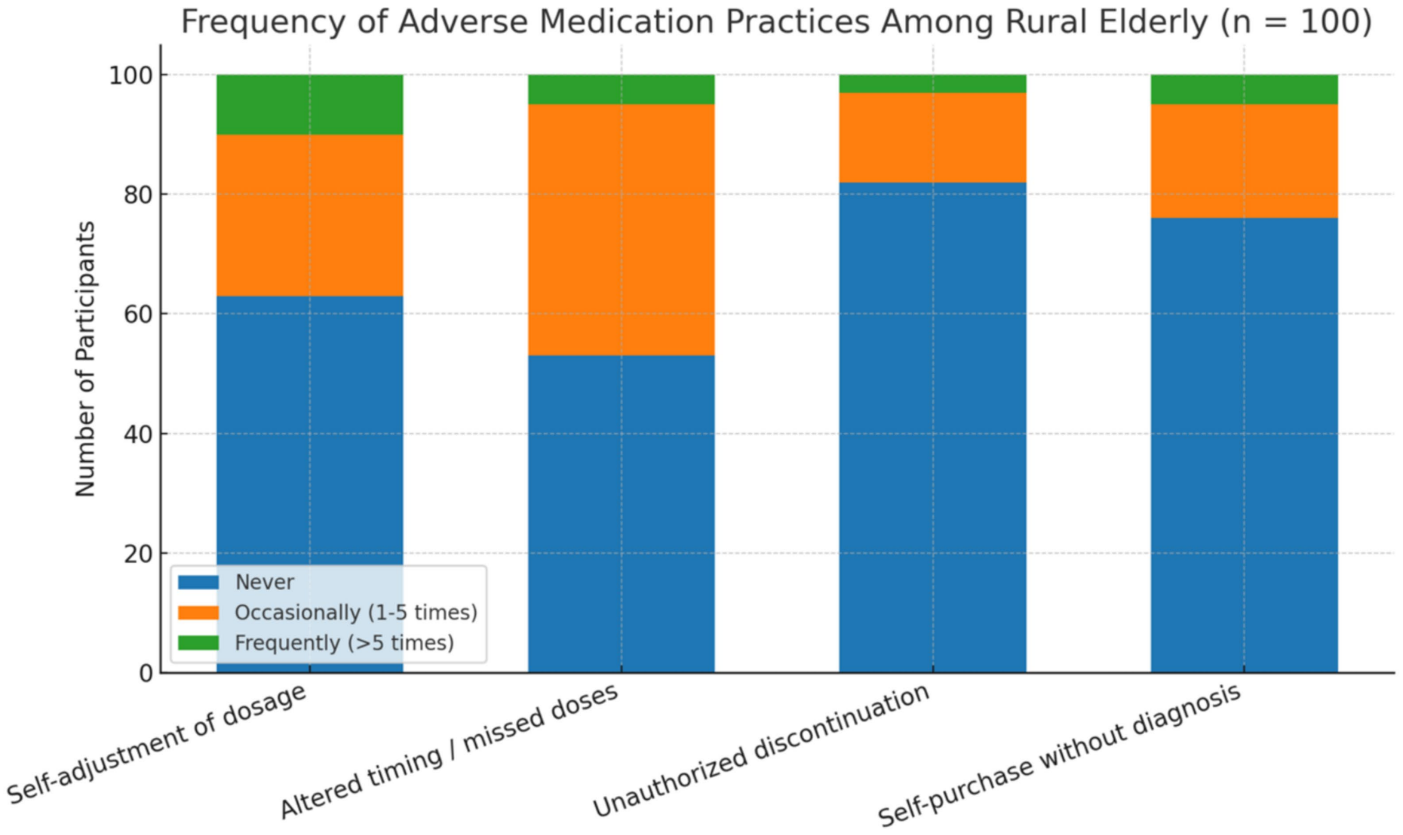
Frequency of self-reported adverse medication practices among rural older participants (*n* = 100). The chart displays the proportion of respondents who reported engaging in each behavior “never,” “occasionally (1–5 times),” or “frequently (>5 times).” Practices included self-adjustment of dosage, altering timing or missing doses, unauthorized discontinuation, and self-purchasing medications without a physician’s diagnosis.

Unsafe medication behaviors were reported by a considerable proportion of participants (Figure 1). Forty-seven (47.0%) altered medication timing or missed doses, 37 (37.0%) self-adjusted dosages, and 24 (24.0%) purchased medication without physician diagnosis. Chi-square analysis indicated that lower education was significantly associated with self-adjusting medication dosage (*p* = 0.013), while lower household income was linked to not consulting a physician before altering medication (*p* = 0.021). Older participants with fewer than 7 years of education were more likely to demonstrate a greater number of unsafe medication behaviors compared to older participants with seven or more years of education, as determined by chi-square analysis (*p* = 0.014).

The physician sample (*n* = 60) included 32 females (53.3%) and 28 males (46.7%) (Table 2). Most were aged 41–50 years (*n* = 29, 48.3%), 29 (48.3%) had completed high school or secondary vocational education, 17 (28.3%) held a junior college degree, and 14 (23.3%) held a bachelor’s degree. Nearly half (*n* = 29, 48.3%) were assistant physicians. Physicians reported high awareness of medication safety (Table 3), with strong agreement on introducing drug names (*n* = 46, 76.7%), indications (*n* = 45, 75.0%), contraindications (*n* = 52, 86.7%), and dosage instructions (*n* = 51, 85.0%) to patients, but fewer engaged patients actively in decision-making.

**Table 2:** Demographic characteristics of rural primary healthcare doctors (*n* = 60).

Characteristic	Category	Percentage (%)
Gender	Male	46
	Female	54
Age group	20–30 years	5
	31–40 years	14
	41–50 years	48
	>51 years	33
Education	High School/Secondary Vocational	48
	Junior College	28
	Bachelor’s Degree	24
	Master’s Degree and above	0
Title	Assistant Physician	48
	Physician	35
	Attending Physician	11
	Associate Chief Physician and above	6

All 60 physician surveys were complete; no missing data were reported.

**Table 3:** Primary healthcare physicians’ cognition of medication safety (*n* = 60).

Content	Strongly agree (%)	Agree (%)	Neutral (%)	Disagree (%)	Strongly disagree (%)
Introduction of drug names to patients	77	20	3	0	0
Introduction of drug indications to patients	75	22	3	0	0
Introduction of drug contraindications to patients	86	12	2	0	0
Introduction of drug usage and dosage to patients	85	15	0	0	0

All 60 physician surveys were complete; no missing data were reported.

Review of 100 EMRs (Table 4) showed that 98 (98.0%) prescriptions scored between 10.5 and 18 points on the MAI, while 2 (2.0%) were rational. PIMs were detected in 12 cases (12.0%), most commonly involving long-acting benzodiazepines and NSAIDs.

**Table 4:** Evaluation of medication rationality (*n* = 100).

MAI score	Number of cases (n)	Percentage (%)
0.5–5 points	1	1
5.5–10 points	1	1
10.5–18 points	98	98
Total (rational drug use rate)	2	2%

## Discussion

The results of this study reveal significant and complex gaps in medication safety for rural older patients in Luzhou City. The high prevalence of polypharmacy (72.0%, *n* = 72, defined as the concurrent use of three or more medications) reflects the substantial burden of chronic conditions in this population, particularly hypertension and cardiovascular disease, which require long-term pharmacological management. Similar prevalence rates have been observed in other Chinese provinces, particularly rural areas with older adult populations coping with multimorbidity and limited access to coordinated care (16, 17).

We found a high prevalence of polypharmacy (72.0%, *n* = 72), defined as the concurrent use of three or more medications. This is comparable to rates reported in other rural Chinese provinces, where chronic conditions such as hypertension and cardiovascular disease are common and require long-term treatment (17, 18). The predominance of antihypertensive and cardiovascular drugs (81.0%) in our cohort reflects the role of cardiovascular morbidity in driving polypharmacy.

Despite widespread use of multiple medications, significant gaps in safe medication knowledge were observed. Less than half (43.0%) of participants consulted a pharmacist, and only 23.0% would discontinue a drug and seek help if an adverse reaction occurred. This limited pharmacovigilance is consistent with findings from other rural regions, where unsafe self-management is linked to low health literacy, poor access to professional advice, and cultural norms favoring self-medication (19, 20). Almost half (47.0%) of participants altered medication schedules or missed doses, and over one-third (37.0%) self-adjusted dosages—behaviors that heighten the risk of treatment failure and adverse drug reactions (21).

The majority of physicians in our sample expressed strong agreement with the need to inform patients about drug names, indications, contraindications, and dosages, suggesting awareness of safe prescribing principles. However, fewer physicians actively engaged patients in decision-making. Similar knowledge–practice gaps have been observed in other countries, often linked to high workload, limited consultation time, and insufficient geriatric pharmacology training. Addressing these barriers is essential for improving patient understanding and adherence.

Our review of 100 EMRs found that 2.0% of prescriptions were rational, and 12.0% contained potentially inappropriate medications (PIMs), primarily long-acting benzodiazepines and NSAIDs. These findings are consistent with international outpatient studies reporting similar PIM prevalence. Given the well-established risks of sedation, falls, renal impairment, and gastrointestinal bleeding in older adults, structured medication review protocols should be prioritized in rural clinical practice.

Lower educational attainment was associated with self-adjusting dosages (*p* = 0.013), and lower household income with changing medication without physician consultation (*p* = 0.021). This reflects broader evidence that socioeconomic disadvantage is a determinant of poor medication adherence and inappropriate use (22). Interventions targeting these high-risk groups, including tailored education and caregiver involvement, could yield substantial benefits.

These findings support several concrete public health actions. First, community-based medication education initiatives—delivered through village clinics or home visits—could directly address the knowledge gaps and self-management risks identified in this population. Second, policy interventions such as mandating continuing education in geriatric pharmacotherapy for rural primary care physicians may strengthen safe prescribing practices. Integrating pharmacists or pharmacy assistants into rural primary healthcare teams, even part-time, could further reduce inappropriate medication use. In terms of health system planning, incorporating electronic medication review tools or mobile health platforms (e.g., SMS reminders or caregiver alerts) may offer scalable solutions to improve adherence and safety in low-resource settings. Together, these interventions could inform a multifaceted public health strategy aimed at reducing medication-related harm among the rural older people. Overall, our findings suggest that improving medication safety in rural older populations will require a multifaceted approach: expanding patient education, enhancing physician communication skills, integrating pharmacists into primary care teams, and implementing regular medication reviews guided by tools such as the MAI and Beers Criteria (23).

### Limitations

This study has several limitations. First, the sample sizes for both older participants (*n* = 100) and physicians (*n* = 60) were relatively small and drawn from a single region, which may limit the external validity and generalizability of the findings to other rural settings in China. Additionally, the use of convenience sampling may introduce selection bias. Second, self-reported data are subject to recall and social desirability bias, although we implemented measures to mitigate these effects. Third, while the survey instruments demonstrated acceptable internal consistency, they were not subjected to full psychometric validation. In future studies, we plan to improve the robustness of the instruments by conducting formal assessments of construct validity, content validity, and test–retest reliability. Expanding to multi-site samples and using probability-based sampling methods would also enhance generalizability and methodological rigor.

### Implications and future directions

These findings underscore the importance of ongoing medication safety education for the rural older people. One way to enhance adherence and decrease use of PIMs could be to improve the environments of primary care providers through geriatric pharmacotherapy training initiatives for physicians and through recruiting clinical pharmacy specialists to review medication profiles routinely.

Additionally, the use of digital health tools, such as mobile reminders for medications, alerts for family caregivers, and tele-pharmacy services, may enable medication management strategies to be implemented more safely in areas of limited access and/or rural areas. Future studies to evaluate these strategies would benefit from being conducted in larger, multisite studies with validated surveys and longitudinal follow-up.

## Conclusion

Overall, our findings suggest that improving medication safety in rural older populations will require a multifaceted approach. Expanding patient education is essential given the clear association between low medication literacy and unsafe behaviors such as self-adjustment of medication regimens and low consultation rates. Enhancing physician communication skills and actively involving patients in treatment decisions may help bridge the observed knowledge–practice gap. Integrating pharmacists into primary care teams could improve medication adherence and reduce inappropriate drug use, particularly in resource-constrained rural settings. Additionally, regular medication reviews guided by validated tools such as the Beers Criteria and Medication Appropriateness Index (MAI) are critical to minimizing the use of potentially inappropriate medications and reducing the risk of adverse drug events. These strategies, particularly when tailored to address socioeconomic disparities, may substantially improve the safety and effectiveness of pharmacotherapy for older patients in rural China.

## Data Availability

The original contributions presented in the study are included in the article/Supplementary material, further inquiries can be directed to the corresponding author.
