# Supplementary material for: A study of medication safety in rural older people in Luzhou City
Source: Front Public Health. 2026 Jan 14;13:1656427. doi: 10.3389/fpubh.2025.1656427 (PMC12848795; doi:10.3389/fpubh.2025.1656427)
Supplement: Supplementary file 1 [file Data_Sheet_1.docx]

Luzhou City Rural Elderly Rational Medication Knowledge Survey Questionnaire

Dear Patient,

Hello! We are a rational medication research team from Luzhou City People’s Hospital. Thank you for taking the time to participate in our questionnaire survey. This study is part of the 2023 Luzhou City Population and Development Research Project (RK202301). The survey is conducted anonymously, and the results will be used solely for academic research and analysis. We strictly guarantee the confidentiality of your personal information. Please feel assured.

Gender:                                    Age:

Education level：

Marital status：

Chronic disease：
Household income:

Types of diseases ( )

A. Hypertension

B. Diabetes

C. Coronary Heart Disease

D. Cerebrovascular Disease

E. Bone/Joint Disease

Number of medications taken ( )

A. 1-2 types

B. 3-4 types

C. More than 5 types

Self-adjusting medication dosage (increase or decrease) ( )

A. Never

B. Occasionally (1-5 times)

C. Frequently (>5 times)

Changing medication time, interval, or missing doses ( )

A. Never

B. Occasionally (1-5 times)

C. Frequently (>5 times)

Stopping medication too quickly or without medical advice ( )

A. Never

B. Occasionally (1-5 times)

C. Frequently (>5 times)

Basis for selecting medications ( )

A. Doctor’s prescription

B. Pharmaceutical advertisements

C. Pharmacist’s recommendation

D. Self-judgment

E. Recommendations from unlicensed practitioners

Purchasing medications from pharmacies without a doctor’s diagnosis ( )

A. Never

B. Occasionally (1-5 times)

C. Frequently (>5 times)

Actively consulting pharmacists about medication usage, dosage, or precautions when purchasing ( )

A. Frequently

B. Sometimes

C. Rarely

Response to adverse drug reactions ( )

A. Unaware of adverse reactions

B. Aware but continued taking the medication

C. Stopped medication and consulted a doctor

D. Stopped medication without further action
