# Supplementary material for: A study of medication safety in rural older people in Luzhou City
Source: Front Public Health. 2026 Jan 14;13:1656427. doi: 10.3389/fpubh.2025.1656427 (PMC12848795; doi:10.3389/fpubh.2025.1656427)
Supplement: Supplementary file 2 [file Data_Sheet_2.docx]

Survey on Rational Medication Knowledge Among Rural Elderly in Luzhou City

Dear Doctor,

Greetings! We are the Rational Drug Use Research Team from Luzhou People’s Hospital. Thank you for taking the time to participate in our survey amidst your busy schedule. This study is part of the 2023 Luzhou Population and Development Research Project (RK202301). The survey is conducted anonymously, and the results will be used solely for academic research and analysis. We strictly guarantee the confidentiality of your personal information. Please answer each question carefully and thoroughly. Your cooperation is greatly appreciated!

Luzhou Rural Doctors’ Knowledge-Attitude-Practice (KAP) Survey Questionnaire

For the following items, please mark "√" under the selected option or fill in your answer accurately. Thank you for your cooperation!

I. Personal Information

Gender:

① Male ② Female

Age:

____________ years old

Education Level:

① High School/Vocational School ② College ③ Bachelor’s Degree ④ Master’s Degree or above

Professional Title:

① Assistant Doctor ② Physician ③ Attending Physician ④ Deputy Chief Physician or above

II. Doctors’ Attitudes Towards Patients’ Medication Safety

Options: Strongly Disagree Disagree Neutral Agree Strongly Agree

1. It is necessary to inform patients about medication names.

2. It is necessary to inform patients about medication indications.

3. It is necessary to inform patients about dosage and administration.

4. It is necessary to inform patients about dietary and drug contraindications.

5. Medication should be the first choice for treatment after illness.

6. Follow-up during medication is essential.

III. Doctors’ Knowledge and Awareness of Patients’ Medication Safety

Options: Yes No Uncertain

1. Do you consider the patient’s age when prescribing medication?

2. Do you consider the patient’s liver/kidney function and organ health?

3. Is prescribing more medications better during treatment?

4. Do you adjust treatment plans proactively if efficacy is poor?

5. Do you advise patients to stop or switch medication if adverse reactions occur?

6. Do you advise patients to abstain from smoking and alcohol during medication?

IV. Doctors’ Practices in Patients’ Medication Safety

Options: Never Occasionally Frequently Always

1. Do you thoroughly explain medication effects and usage to patients?

2. Do you clearly explain dosage and treatment duration to patients?

3. Do you adjust medication types based on the patient’s condition?

4. Do you adjust dosage and duration based on the patient’s condition?

5. Do you report adverse drug reactions promptly?

6. Do you consult guidelines or drug manuals for unfamiliar medications?

End of Survey

Thank you again for your cooperation! Wishing you success in your work and happiness in life!
